# A mathematical model to guide antibiotic treatment strategies

**DOI:** 10.1186/1741-7015-10-90

**Published:** 2012-08-13

**Authors:** Albert Sotto, Jean-Philippe Lavigne

**Affiliations:** 1 Institut National de la Santé et de la Recherche Médicale, U1047, Université Montpellier 1, UFR de Médecine 30908 Nîmes Cedex 08, France; 2 Service des Maladies Infectieuses et Tropicales, CHU Caremeau, 30029 Nîmes Cedex 09, France

**Keywords:** antibiotic resistance, mathematical models, multidrug resistance

## Abstract

Over the past few decades, the emergence of multidrug resistance (MDR) to antibiotics in bacteria has led to major difficulties in the management of infected patients. At present, there is a serious lack of development of new antibacterial agents. Mathematical models are one approach to understand how antibiotic usage patterns may be optimized. However, the classical approach to modeling the emergence of MDR relies on the simplifying assumption that resistance is acquired at a constant rate. In their model, Obolski and Hadany introduce the notion of horizontal gene transfer and stress-induced mutation, with antibiotics constituting an environmental stressor of particular relevance. Finally, from this complex mathematical model, the authors propose predictions for minimizing MDR in bacteria depending on strategies of antibiotic treatment.

Please see related article: http://www.biomedcentral.com/1741-7015/10/89

## Background

Over the past few decades, the emergence of bacteria resistant to multiple antimicrobial agents has led to major difficulties in the management of infected patients. This has required significant changes in our approaches to the use of antibiotics and should lead to new ways of thinking about the production of effective antibacterial drugs. Unfortunately, at present, there is a serious lack of development of new antibacterial agents, specifically concerning Gram-negative bacilli. Programs, supported by the medical community and government organizations, have been implemented to limit the emergence of multidrug resistance (MDR) to antibiotics: reducing the inappropriate use of antibiotics (not treating colonization; antibiotic use is best suited to the clinical situation) and improving standards of hygiene to prevent crosstransmission. It is clear that these measures were not sufficient to stop the process [1]. Other approaches are possible, such as modeling the emergence of MDR. In a study by Obolski and Hadany published in *BMC Medicine*, the authors' objective was to explore the impact of genetic variation induced by antibiotic stress on the spread of MDR in a hospital unit [2]. The purpose of such models is to understand how antibiotic usage patterns may be optimized.

## Clinical importance of the mathematical model

In their rationale, Obolski and Hadany highlighted the importance of three different strategies (cycling, mixing and combining) of antibiotic prescribing to minimize the emergence of MDR. In the cycling regime, antibiotic treatment is the same for all patients at a given time and the treatment is periodically switched. In the mixing strategy, each patient receives a randomly selected drug. Combining involves administering several antibiotic treatments to each patient. The authors noted the ability of each strategy to prevent the risk of emergence of MDR, indicating that more antibiotics were used in combining than in mixing or cycling. They emphasize that the classical approach to modeling the emergence of MDR relies on the simplifying assumption that resistance is acquired at a constant rate, ignoring recent data on the effect of environmental stress on mechanisms of acquired resistance. This erroneous assumption could be the cause of frequent reservations in the conclusions of these models. Moreover, the actual effect of a single intervention in a complex situation is often difficult to grasp [3]. The originality in the model used by Obolski and Hadany is to introduce the notion of horizontal gene transfer (HGT) and stress-induced mutation (SIM), antibiotics constituting an environmental stressor of particular relevance. The authors justify this hypothesis by showing examples of stress-induced HGT and SIM for various microorganisms that may be encountered in community-acquired infections (*Streptococcus pneumoniae*, *Escherichia coli*), nosocomial (*Pseudomonas aeruginosa*, *E. coli*) or epidemic infections (*Vibrio cholerae*). The model is used, but adapted to integrate the concepts of stress-induced HGT and SIM [4]. The authors also introduce some constraints in the model: (i) the bacterial pathogens are assumed to accompany other ailments and not be the main reason for hospitalization, (ii) since the bacterial infection is not the main reason of hospitalization patients leave the hospital or die at a rate proportional to their frequency, (iii) hospital occupancy is kept constant, (iv) there are no double-resistant bacteria in the hospital initially, and that their frequency in the general population is negligible. Concerning the SIM, they also took into consideration modeling the relative persistence of antibiotic resistance when there is no direct antibiotic use. The introduction of different level assumptions (antibiotic resistance persistence and stress-induced mutation) has had different consequences in terms of the proportion of infected patients and emergence of resistance. Indeed, the latter two parameters may vary either in the same direction or in the opposite direction in functions of the hypotheses. Another mechanism of acquisition of antibiotic resistance studied in this model was HGT. The latter was studied in defining an encounter rate between bacteria. The authors also considered situations in which the bacteria were or were not under antibiotic stress. They developed the notion of time required for the cellular mechanisms to induce or repress HGT. They defined an extreme situation where the HGT rates of bacteria transmitted from patient A to patient B depend only on the stress of the bacteria experienced while residing in patient A. Thus, assessing the effects of different antibiotic regimens with respect to the encounter rates between bacteria is complex.

Finally, from this complex mathematical model, based on the important concepts of SIM and HGT, several conclusions can be proposed. The authors have shown the relationship between stress-induced genetic variation and the emergence of double resistance. Combining performs very poorly in inhibiting double resistance emergence when genetic variation is stress induced. Cycling is the preferred strategy with respect to the acquisition of resistance through SIM. When there is stress-induced HGT, cycling and mixing are the favored strategies. The speed of change in HGT frequencies in response to antibiotic stress determines the choice of strategy between mixing and cycling. The authors note that their predictions hold even for very mild increases of HGT and mutation rates under antibiotic stress in comparison with those described in the literature. However, the cited publications were referring to *in vitro *studies [5-8]

## Future directions and conclusions

The present model has shown that combining was slightly more efficient than mixing and cycling with regard to minimizing infection. Regarding double resistance emergence, both mixing and cycling were more efficient than combining when variation is stress induced.

The authors have described the limits of their work: they did not consider the possible fitness cost of antibiotic resistance or the influence of stochastic events (epidemic outbursts of bacteria and extinction of rare bacterial strains for long periods of time, human errors in the form of dosage errors, lack of compliance to hospital guidelines) on the dynamics. Other limiting factors were also mentioned such as patient age, relative efficiency of the different antibiotics and drug interactions. In our opinion, other limits could also have been mentioned: the problems of non-adherence, self-medication, outpatient antibiotic use, and the state of immunosuppression of the patient treated.

Mathematical models such as that built by Obolski and Hadany are an important step in regulating the consumption of antibiotics. The originality in this model is the study of stress-induced genetic variation mechanisms on resistance emergence and minimization of infection. However, in the literature, it is usual that models consider only a single pathogen or a single mechanism of resistance acquisition and it is not clear that they can be applied to other clinical situations. At the same time, with the aim to try to stop the continuing spiral of resistance it is necessary to develop other research programs: campaigns to educate the public and healthcare workers [9], novel avenues for drugs targeting against infectious agents [10,11], the development of phage strategies [12,13] and new conjugate vaccines [14], the use of new agents such as probiotics or antivirulence drugs [15,16], and the use of new diagnostic tools to differentiate colonization from infection [17]. Only with a multidisciplinary approach will win the fight against MDR bacteria.

## Competing interests

The authors declare that they have no competing interests.

## Authors' contributions

Both authors contributed equally to the preparation of the manuscript, and both authors approved the final version to be published.

## Authors' information

AS (MD, PhD) is head of the Infectious Diseases Department of the University Hospital of Nîmes. He is currently affiliated with, and conducting research at, the National Institute of Health and Medical Research, U1047, Faculty of Medicine, Montpellier 1 University, Montpellier, France.

Since 2011, JPL (MD, PhD) has been University Professor of Bacteriology at the University Montpellier 1 and in University Hospital of Nîmes. He leads a team in INSERM U1047, which works on bacterial virulence notably concerning the virulence of MDR bacteria.

## Pre-publication history

The pre-publication history for this paper can be accessed here:

http://www.biomedcentral.com/1741-7015/10/90/prepub
